# Improved Photometric Standards and Calibration Procedures at NIST

**DOI:** 10.6028/jres.102.022

**Published:** 1997

**Authors:** Yoshihiro Ohno

**Affiliations:** Optical Technology Division, National Institute of Standards and Technology, Gaithersburg, MD 20899-0001

**Keywords:** calibration, candela, illuminance, integrating sphere, lumen, luminance, luminous flux, luminous intensity, lux

## Abstract

NIST has recently established a detector-based luminous intensity unit (candela, cd), which is derived from the NIST absolute cryogenic radiometer. Subsequently, the luminous flux unit (lumen, lm) and the luminance unit (cd/m^2^) have been established based on the detector-based candela, and now all the NIST photometric units are tied to the cryogenic radiometer. The illuminance unit is realized and maintained on five standard photometers. The large dynamic range of the standard photometers eliminates the need for maintaining many working standard lamps of various wattages. The luminous intensities of lamps are determined from the illuminances measured with these photometers and the distances measured with a linear encoder system. Transfer photometers and illuminance meters are calibrated by direct comparison with the standard photometers with no distance measurements involved. The luminous flux unit is realized using an absolute integrating sphere method newly developed at NIST. The luminance unit is realized on an integrating sphere source, which is used for calibration of other luminance sources and luminance meters. These detector-based methods have made it possible to reduce the uncertainties of photometric calibrations and to provide more varieties of photometric calibration services at NIST.

## 1. Introduction

The candela is one of the SI (Systéme International) base units, and hence the basis for all of the photometric quantities. The candela was first defined by the CGPM (Conférence Générale des Poids et Mesures) in 1948 based on the radiation from molten platinum at the temperature of solidification. The candela was redefined by the CGPM in 1979 so that one candela is equal to 1/683 watt per steradian for monochromatic radiation at 555 nm [1]. Since this redefinition, it has been possible to derive the candela from radiometric scales with various methods.

**Fig. 1:**
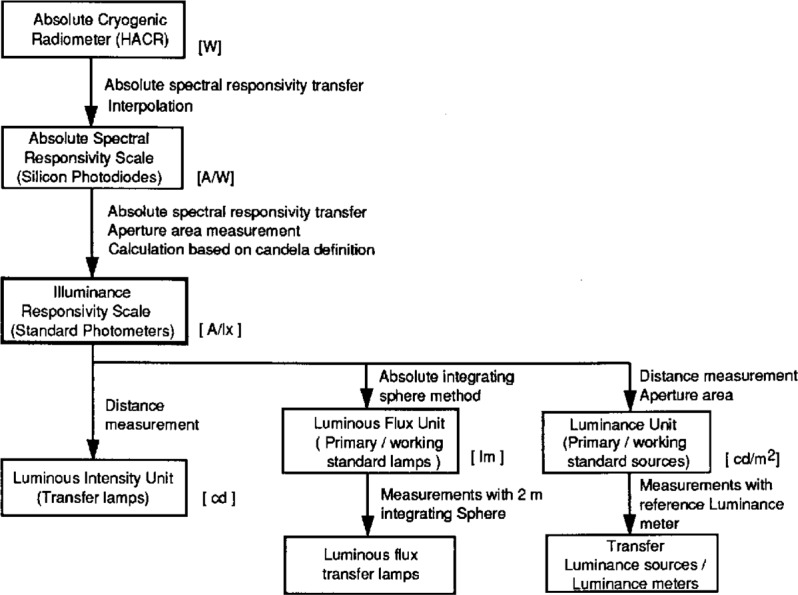
Block diagram showing the realization and maintenance of NIST photometric units.

At NIST, until 1991, the luminous intensity unit (candela) was based on the NIST spectral irradiance scale [2], which was based on a gold-point blackbody. In 1992, a new luminous intensity unit was realized based on an absolute cryogenic radiometer [3, 4]. The realization and maintenance of the photometric units at NIST are depicted in Fig. 1. The NIST cryogenic radiometer [5] (called HACR for High Accuracy Cryogenic Radiometer) acts as the absolute radiometric base at the top of the chain. The radiometer is cooled to 5 K by liquid helium and works on the principle of electrical substitution. Based on laser beam measurements with the HACR at several wavelengths, the NIST spectral responsivity scale [6] is realized on silicon photodiode light-trapping detectors. The NIST candela is realized and maintained via a group of five standard photometers which are characterized for spectral responsivity, and calibrated for illuminance responsivity in the unit A/lx. The standard photometers embody the NIST illuminance unit.

The units of luminous intensity, luminous flux, and luminance are derived from the illuminance unit. The NIST luminous flux unit is realized with a new method using a specially designed integrating sphere system introduced in 1995. The luminance unit is realized based on the luminous intensity and aperture area measurements on a reference integrating-sphere source. The details of the realization of these photometric units and resulting improvements of photometric calibrations at NIST are described in this paper.

## 2. NIST Candela

### 2.1 Principle of the Detector-Based Candela Realization

**Fig. 2:**
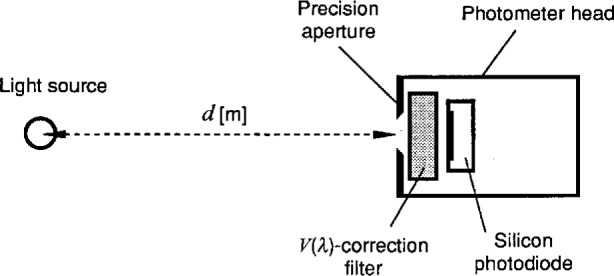
Schematic diagram depicting the geometry for the detector-based candela realization.

A standard photometer consists of a silicon photodiode, a filter that spectrally matches the CIE (Commission Internationale de l’Eclairage) spectral luminous efficiency function for photopic vision (called *V*(*λ*) function), and a limiting aperture, as shown in Fig. 2. When the absolute spectral responsivity *s*(*λ*) [A/W]^1^ of the photometer is measured, the photometric responsivity *R*_v,f_ [A/lm] of the photometer is given by

$$R_{v,f}=\frac{\int_{\lambda }P\left(\lambda \right)s\left(\lambda \right)d\lambda }{K_{m}\int_{\lambda }P\left(\lambda \right)V\left(\lambda \right)d\lambda },$$ (1)

where *P*(*λ*) is the spectral power distribution of light to be measured, *V*(*λ*) is the spectral luminous efficiency function, and *K*_m_ is the maximum spectral luminous efficacy (683 lm/W). Usually a Planckian radiation at 2856 K (CIE Illuminant A) is used for *P*(*λ*). If the responsivity *R*_v,f_ is uniform over the aperture, and with the aperture area *S* known, the illuminance responsivity *R*_v,i_ [A/lx] of the photometer is given by

$$R_{v,i}=S\cdot R_{v,f}.$$ (2)

When the photometer is used to measure the illuminance from a light source (a 2856 K Planckian source and regarded as a point source), the luminous intensity *I*_v_ [cd] of the source is given by

$$I_{v}=d^{2}\cdot y/R_{v,i},$$ (3)

where *d* is the distance from the light source to the aperture surface of the photometer and *y* is the output current of the photometer.

### 2.2 Design of the NIST Standard Photometers

**Fig. 3:**
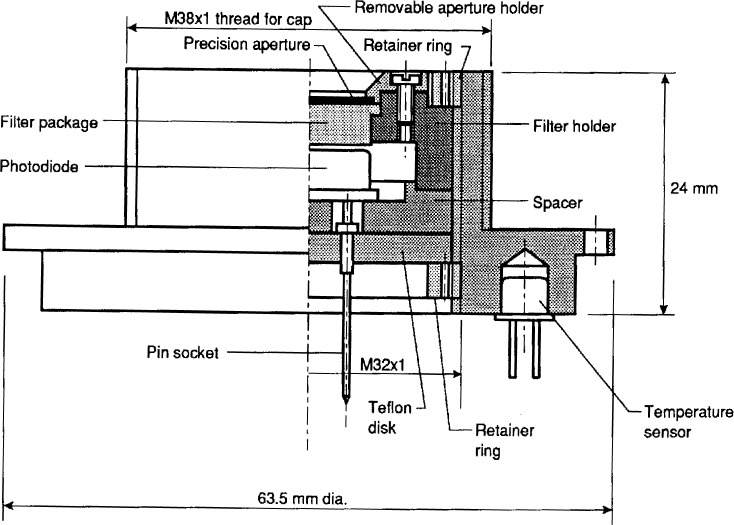
Cross section of the NIST standard photometer.

Figure 3 shows the design of the NIST standard photometers. A silicon photodiode, a *V*(*λ*)-correction filter, and a precision aperture of 3 mm diameter are mounted in the front piece of a cylindrical housing. The silicon photodiode has a sensitive area of 0.3 cm^2^. The *V*(*λ*)-correction filter is made of several layers of glass filters. Since the characteristics of the filter and photodiode change with temperature, a temperature sensor is installed in the front piece of the housing to compensate for the variation of the photometer temperature. Under this front piece, an electronic assembly containing a current-to-voltage converter circuit having a high sensitivity and a wide dynamic range is built in to minimize noise. The circuit has a switchable gain setting from 10^4^ to 10^10^. This high sensitivity feature allows precise measurement of *s*(*λ*) even in the wings of the *V*(*λ*) curve.

### 2.3 Characterization and Calibration of the NIST Standard Photometers

The spectral responsivity *s*(*λ*) of each photometer is measured with the NIST Spectral Comparator Facility [6]. The photometer aperture is underfilled with a beam of 1 mm diameter from the monochromator. The responsivity of the photometer is also mapped over the entire area of the precision aperture at several wavelengths. From the mapping data, the ratio of the average responsivity over the aperture to the responsivity at the center of the aperture is calculated and applied in the responsivity calculation. The areas of the apertures were measured by the Precision Engineering Division of NIST with a relative expanded uncertainty (with a coverage factor of *k* = 2 and thus a two standard deviation estimate) of 0.1 %.

**Fig. 4:**
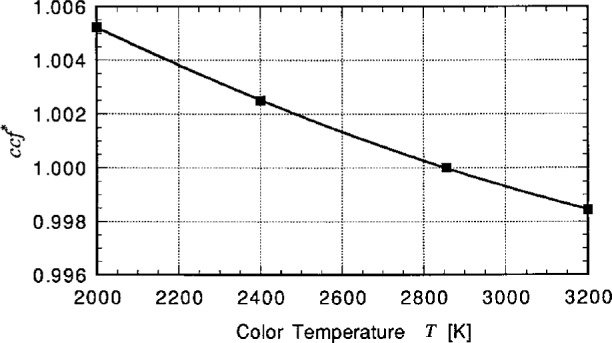
A curve of the form ccf* = *M*_0_ + *M*_1_ *T* + *M*_2_ *T*^2^ fitted to the four points with the result *M_0_* = 1.0253, *M*_1_ = − 1.279.3 × 10^−5^ [K^−1^], *M*_2_ = 1.273 × 10^−9^ [K^−2^].

The illuminance responsivity *R*_v,i_ of the photometers is calibrated for CIE Illuminant A. If the photometer is used to measure sources other than Illuminant A, the responsivity *R*_v,i_ is recalculated with the spectral power distribution of the test source *P*(*λ*) entered in Eq. (1). For convenience in measuring incandescent lamps of various distribution temperatures, spectral mismatch correction factors ccf* (*R*_v,i_ for Illuminant A, divided by *R*_v,i_ for the test source) are obtained and fitted to a polynomial function. An example of the function is shown in Fig. 4. The photometer signal to be corrected is multiplied by ccf*.

The photometers are characterized for temperature dependence. The temperature coefficients of the NIST photometers are measured to be – 0.088 %/°C. Whenever the photometers are used, corrections are made using the temperature sensor signal and the temperature coefficient.

The linearities of the photometers are measured using a beam conjoiner device [7]. The photometers are found to have a linear response over an output currentrange of 10^−10^ A to 10^−4^ A. This corresponds to an illuminance range of 10^−2^ lx to 10^4^ lx. This means that the photometers can be used to measure a luminous intensity as low as 10 mcd at 1 m, and as high as 10^5^ cd at 3 m, without increasing the relative uncertainty significantly.

**Fig. 5:**
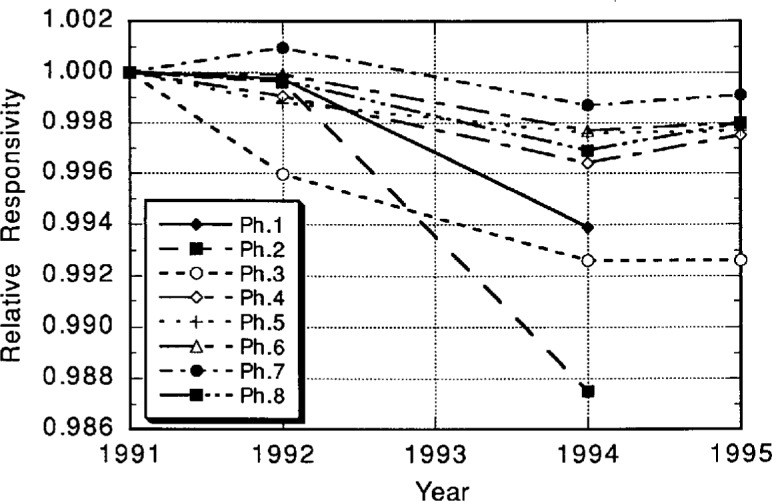
Drift of the illuminance responsivity of the NIST standard photometers over a four year period.

The responsivities of photometers in general are subject to drift over time. The NIST standard photometers are calibrated on an annual basis. Initially eight photometers were used. The drift of the illuminance responsivity of the eight standard photometers over a 4 year period is shown in Fig. 5. While photometers 1, 2, and 3 showed significant changes, photometers 4 through 8 showed drifts of less than 0.1 % per year. Since 1995, only these five photometers have been used to maintain the unit. Note that these results include the uncertainty of the illuminance unit realization.

The relative expanded uncertainty (*k* = 2) of the NIST illuminance unit is 0.38 %, which is an improvement by a factor of two over the previous source-based illuminance unit. The details of this uncertainty budget are described in Ref. [2].

### 2.4 Luminous Intensity Calibration

At NIST, the luminous intensities of test lamps were previously calibrated against luminous intensity standard lamps. Working standard lamps operating at several different power levels were used to compare lamps operating at similar power levels, to avoid linearity errors in the detector system. The burning time of the primary and working standard lamps was strictly limited in order to minimize the aging of the lamps. Additional components of uncertainty were included when the scale was transferred from the primary standard lamps to the working standard lamps.

By utilizing the detector-based method, the luminous intensities of lamps in a wide range of intensity are directly calibrated by the standard photometers because of their wide domain of linearity. The standard photometers (primary standards) can be used in routine calibrations since they do not age with use as lamps tend to do. The relative expanded uncertainty (*k* = 2) of luminous intensity calibrations at NIST is typically 0.52 %, which includes the uncertainty of the illuminance unit (0.38 %), the long-term drift of the standard photometers (0.26 %), the reproducibility of test lamps (0.2 %), and other factors. A photometric bench with an accurate length scale remains essential for luminous intensity calibrations. The need for luminous intensity standard lamps, however, can be eliminated in most cases by replacing standard lamps with standard photometers. A method for determining the color temperature of the test lamp (incandescent lamp) is required for the highest accuracy measurements. Greater care must be taken to minimize stray light, since it is not canceled out as it is in the lamp-to-lamp substitution measurements.

### 2.5 Illuminance Meter Calibration

With the previous source-based method, illuminance calibrations rely on the luminous intensity of a standard lamp, the inverse square law, and accurate measurements of distance. Alignment of the standard lamp is critical, and the departure from the inverse-square law at short distances is a large uncertainty factor.

The detector-based method has a greater advantage in the calibration of illuminance meters and transfer photometers. At NIST, illuminance meters and photometers are calibrated by direct substitution with the standard photometers by placing them on the same illuminated plane. In such substitution methods, many uncertainty factors are canceled out. There is no need for distance measurements. The lamp alignment and the departure from the inverse square law are no longer critical factors. A working lamp of known color temperature (normally 2856 K) is used. The short-term stability of the working lamp is important, but its burning time and aging characteristics are not of concern.

The relative expanded uncertainty (*k* = 2) of the illuminance meter calibration at NIST is typically 0.50 %, which includes the uncertainty of the illuminance unit (0.38 %), long-term drift of the standard photometers (0.26 %), random variations in transfer measurement (0.1 %), and other factors. This value does not include uncertainty factors inherent to the illuminance meter under test.

## 3. NIST Lumen

### 3.1 Principle of the Absolute Integrating Sphere Method

A new method to realize a luminous flux unit has been developed at NIST using a special integrating sphere instead of a goniophotometer. The basic principle of this method (the absolute integrating sphere method) is to calibrate the total flux of a lamp inside the sphere against the known amount of flux introduced from a source outside the sphere through an opening. This method was first proposed through a theoretical analysis using a computer simulation technique [8], then experimentally verified [9], and was actually applied to the realization of the NIST luminous flux unit in 1995 [10].

**Fig. 6:**
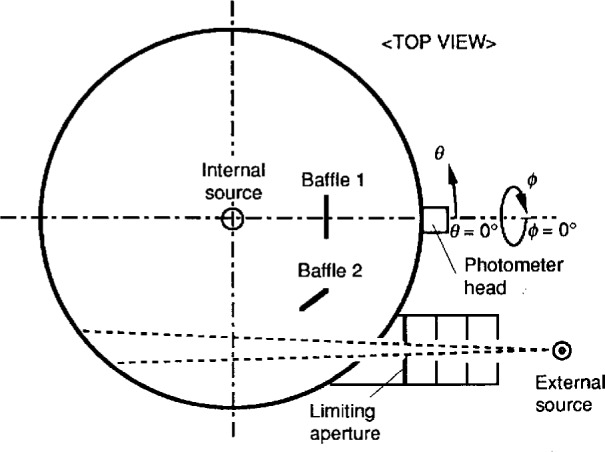
Schematic diagram showing the setup for the absolute integrating sphere method.

Figure 6 shows the arrangement for the absolute integrating sphere method. The flux from the external source is introduced through a calibrated aperture placed in front of the opening. The internal source, a lamp to be calibrated, is mounted in the center of the sphere. Two baffles are used to shield the detector and the opening from direct illumination by the internal source. The detector is exposed to the “hot spot” (the first reflection of the flux introduced from the external source) in order to equalize the sphere responsivity for the internal source and that for the external source. Baffle 2 is aligned so that neither surface is viewed by the detector.

In this method, the external source and the internal source are operated alternately, and the total luminous flux *Φ*_i_ of the internal source is obtained by comparison to the luminous flux introduced from the external source as given by,

$$\Phi_{i}=c_{f}E_{a}Ay_{i}/y_{e},$$ (4)

where *E*_a_ is the average illuminance [lx] from the external source over the limiting aperture of known area *A*, *y*_i_ is the detector signal for the internal source, and *y*_e_ is the detector signal for the external source. The quantity *c*_f_ is an important correction factor for various non-ideal behaviors of the integrating sphere. The response of the integrating sphere is not uniform over the sphere wall due to baffles and other structures inside the sphere, and also due to nonuniform reflectance of the sphere wall due to contamination. The light from the external source is incident at 45° while the light from the internal source is normal. When the incident angle is different, the diffuse reflectance of the sphere coating changes, which affects the sphere responsivity. When the spectral power distribution of the internal source is different from that of the external source, a spectral mismatch error occurs. All these corrections are made to determine the correction factor *c*. A self-absorption correction is not necessary if the internal source to be calibrated stays in the sphere when the external source is measured.

### 3.2 Correction for the Spatial Nonuniformity Errors

The spatial responsivity distribution function (SRDF) of the sphere, *K*(*θ*,*ϕ*), is defined as the sphere response for the same amount of flux incident on a point (*θ*,*ϕ*) of the sphere wall or on a baffle surface, relative to the value at the origin, *K*(0,0). *K*(*θ*,*ϕ*) can be obtained by measuring the detector signals while rotating a narrow beam inside the sphere. The rotating lamp must be insensitive to burning position. *K*(*θ*,*ϕ*) is further normalized for the sphere response to an ideal point source. The normalized SRDF, K*(*θ*,*ϕ*), is defined as

$$K*\left(\theta ,\phi \right)=4\pi K\left(\theta ,\phi \right)/\int_{\phi =0}^{2\pi }\int_{\theta =0}^{\pi }K\left(\theta ,\phi \right)\sin\theta \;d\theta \;d\;\phi .$$ (5)

**Fig. 7:**
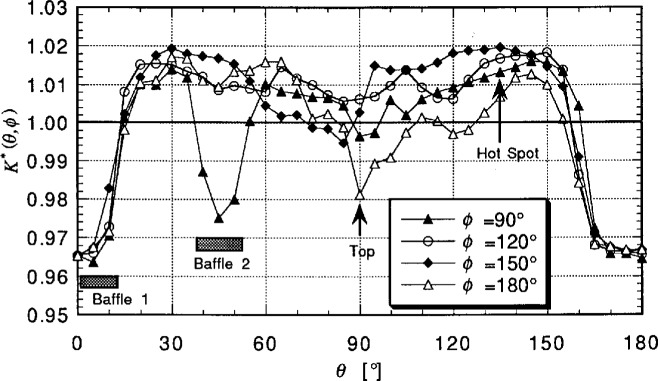
The Spatial Response Distribution Function (SRDF) of the NIST integrating sphere.

Figure 7 shows the SRDF of the NIST integrating sphere. Using K*(*θ*,*ϕ*), the spatial correction factor *scf*_e_ for the external source with respect to an isotropic point source is given by

$$scf_{e}=1/K*\left(\theta_{e}/\phi_{e}\right),$$ (6)

where (*θ*_e_,*ϕ*_e_) is the point at which the center of the area illuminated by the external source is located. The spatial correction factor *scf*_i_ for the internal source with respect to a point source is given by

$$scf_{i}=1/\int_{\phi =0}^{2\pi }\int_{\theta =0}^{\pi }I*\left(\theta ,\phi \right)K*\left(\theta ,\phi \right)\;\sin\theta \;d\theta \;d\phi ,$$ (7)

where I*(*θ*,*ϕ*) is the normalized luminous intensity distribution of the internal source given by

$$I*\left(\theta ,\phi \right)=I_{\text{rel}}\left(\theta ,\phi \right)/\int_{\phi =0}^{2\pi }\int_{\theta =0}^{\pi }I_{\text{rel}}\left(\theta ,\phi \right)\;\sin\theta \;d\theta \;d\phi ,$$ (8)

where *I*_rel_(*θ*,*ϕ*) is the relative luminous intensity distribution of the internal source.

A goniophotometer is not necessarily required to obtain *I*_rel_(*θ*,*ϕ*). Most of the total luminous flux standard lamps have fairly uniform angular intensity distributions and *scf*_i_ can be assumed unity (when the sphere reflectance is relatively high). Even when Eq. (8) is applied, only relative intensity distribution is necessary, and its accuracy is not critical. For example, the data for a group of lamps of the same type can be represented by one lamp. Once the distribution data are taken, they are used for the lifetime of the lamps.

### 3.3 Realization of the NIST Lumen

The NIST 2 m integrating sphere has been modified to have the geometry shown in Fig. 6. The sphere is coated with barium sulfate paint with a reflectance of approximately 97 % in the visible region. An opening of 10 cm diameter was cut at a position 45° away from the detector. Baffle 1 (20 cm in diameter) is located 50 cm from the sphere center. Baffle 2 (15 cm in diameter) is located 60 cm from the sphere center.

The detector is a *V*(λ)-corrected photometer with an opal diffuser (20 mm diameter) attached in front. It has a built-in transimpedance amplifier with gain settings from 10^4^ V/A to 10^10^ V/A. A built-in temperature sensor allows corrections for the photometer temperature drift. The linearity of the detector was measured to be constant over a flux range of 10^−1^ lm to 10^5^ lm to within 0.05 %.

A 1000 W frosted FEL type quartz halogen lamp operated at 2856 K is used as the external source. The lamp is placed at 70 cm from the limiting aperture, introducing a flux of approximately 2.7 lm or approximately 4.2 lm through a stainless steel aperture of 40 mm or 50 mm diameter and 3 mm thickness. The areas of the apertures were determined by the NIST Fabrication Technology Division with a relative expanded uncertainty (*k* = 2) of 0.03 %. The aperture is placed as close to the opening as possible to minimize diffraction losses. The illuminance distribution over the aperture area is measured by spatially scanning a cosine corrected photometer to determine the average illuminance correction factor *k*_a_, which is the ratio of the average illuminance *E*_a_ to the illuminance on the aperture center *E*_c_. The NIST standard photometers are used to determine the illuminance *E*_c_.

The SRDF, *K*(*θ*,*ϕ*), is measured by rotating a beam source which is burning position insensitive. A 6 V (1.2 W) vacuum incandescent lamp equipped with a reflector (40 mm diameter) and a cylindrical hood (100 mm long) are used. The beam angle is about 10°. The SRDF measurements are made at 5° intervals for *θ* and 30° intervals for *ϕ*. Figure 7 shows part of the SRDF curves of the NIST integrating sphere. The polar coordinates (*θ*,*ϕ*) in the graph have their origins in the position of the photometer head as illustrated in Fig. 6. From these data, *scf*_i_ for the internal source (a 40 W opal lamp) and *scf*_e_ for the external source were calculated.

A group of twelve 40 W opal-bulb incandescent lamps, operated at 2730 K, is calibrated to serve as the luminous flux primary standards. At the same time, eight 60 W inside frosted incandescent lamps, operated at 2740 K, are also calibrated to serve as working standards. Routine calibrations for luminous flux are performed using these working standard lamps. The working standard lamps are recalibrated at every 10 h of total operating time. Spectral mismatch correction factors ccf* were calculated for these lamps, and corrections were applied. The self-absorption of each lamp was also measured and corrections were made. The relative expanded uncertainty (*k* = 2) of the NIST luminous flux unit is 0.53 %. The details of the uncertainty budget are discussed in Ref. [10].

### 3.4 Total Luminous Flux Calibration

In the past, a strict substitution method was employed at NIST. Working-standard lamps of 500 W, 200 W, 100 W, and six types of miniature lamps ranging from 400 lm to 6 lm were maintained for luminous flux calibration [2]. Additional uncertainty components were added when these working standard lamps were calibrated from one group to another, starting from the 300 W primary standard lamps. The relative expanded uncertainties (*k* = 2) of working standard lamps ranged from 1.0 % to 1.9 %.

With the absolute integrating sphere method described above, the luminous flux unit is realized on an annual basis. The primary standards are used only to cross check the consistency of the unit. Working standard lamps are annually calibrated directly by the absolute integrating sphere method with the same uncertainty as primary standard lamps. With the use of a photometer having a wide range of linearity, and with the spatial and spectral correction techniques recently developed, test lamps ranging from 10^−1^ lm to 10^5^ lm can be measured in the 2 m sphere against one type of working standard lamp. Although this is still a substitution method, many types of working standard lamps are no longer needed.

In routine calibrations, corrections are always made for self-absorption, spectral mismatch errors, and photometer temperature variations. For the spectral mismatch correction, the spectral power distributions or the color temperatures of test lamps are measured. A spatial nonuniformity correction is made only when special types of lamps having directional intensity distributions are calibrated.

With the new calibration procedures, the uncertainty of luminous flux calibrations has been reduced significantly. The relative expanded uncertainty (*k* = 2) of the luminous flux calibration of incandescent lamps is now typically 0.8 %, which includes the uncertainty of the NIST luminous flux unit (0.53 %), aging of the working standard lamps between calibrations (0.30 %), transfer from working standards to test lamps (0.5 %), and reproducibility of test lamps (0.2 %).

## 4. NIST Luminance Unit

A luminance unit is commonly established using a white reflectance standard or a transmitting diffuser, such as opal glass, illuminated by a luminous intensity standard lamp. The uncertainty of such a luminance unit includes that of the standard lamp, that of the reference material, and other factors such as stray light. Opal glass is particularly sensitive to stray light coming from both directions.

**Fig. 8:**
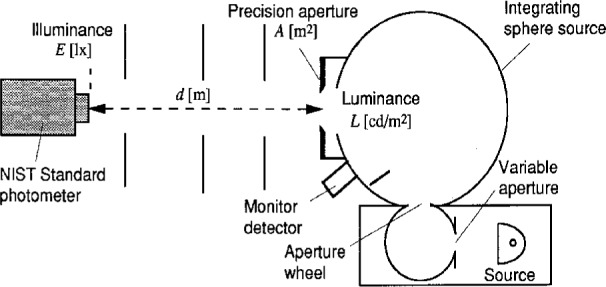
Configuration for NIST luminance unit realization.

With the use of the standard photometers described above, a detector-based luminance unit is realized at NIST on a reference integrating-sphere source. Figure 8 shows the configuration. The sphere source is 15 cm in diameter and has a *V*(*λ*)-corrected monitor detector on the sphere wall. The source has a double sphere structure, the large sphere being irradiated by an intermediate 5 cm sphere which is irradiated by a quartz halogen lamp. The sphere source is operated at 2856 K by a constant-current power supply. Two precision apertures of approximately 8 mm diameter are attached alternately to the exit port (50 mm diameter) of the sphere source. The sphere source and the photometers are placed on the photometric bench in a light tight box to minimize stray light errors. The illuminances at 2 m from the sphere source are measured by the NIST standard photometers. The distance is measured with a linear encoder system. The sphere source is stabilized for 60 min before calibration since the responsivity of the monitor detector drifts as the sphere warms up. The average luminance *L* [cd/m^2^] over the aperture plane is simply determined from the illuminance *E*, the distance *d*, and the aperture area *A*, as given by

$$L=E\;d^{2}/A.$$ (9)

A geometrical correction factor determined from the areas of the aperture and the photometer sensitive surface is calculated to be negligible in the given geometry. The diffraction loss with the aperture is also calculated to be negligible. When the luminance *L* is determined, the monitor detector signal is recorded and the monitor detector responsivity *R*_L_ is determined. The monitor detector views a part of the sphere wall across from the exit port and represents the luminance on the exit port regardless of the aging of the lamp and the interreflection effect by the aperture.

For routine calibrations, the precision aperture is removed and the sphere port can be completely opened (50 mm diameter) or equipped with another aperture (25 mm diameter). In this case, the monitor detector value still represents the luminance for the 8 mm aperture. Therefore, when an average luminance over the entire exit port (or 25 mm aperture) is needed, the ratio of the center luminance (8 mm) to the average luminance is obtained from the spatial distributions of luminance over the exit port.

The luminance unit is realized annually using these procedures and maintained via the responsivity *R*_L_ of the monitor detector. The color temperature of the sphere source is also recalibrated annually. The unit is transferred to a reference luminance meter used to cross check the sphere source. The relative expanded uncertainty (*k* = 2) of the NIST luminance unit is 0.46 %, which includes the uncertainty of the NIST illuminance unit (0.38 %), long-term drift of the standard photometers at the time of use (0.1 %), the aperture area (0.10 %), alignment and distance measurements (0.05 %), reproducibility of the sphere source (0.16 %), and other factors.

## 5. Conclusion

The luminous intensity unit (candela, cd), luminous flux unit (lumen, lm), and the luminance unit [cd/m^2^] have been established utilizing the detector-based method. All the NIST photometric units are now based on the absolute cryogenic radiometer. The uncertainties of these NIST photometric units have been significantly reduced. Rather than depending on the long-term stability of reference artifacts, these photometric units are realized on an annual basis in order to keep their uncertainties to a minimum.

The detector-based method is also utilized in the photometric calibrations of the luminous intensities of lamps, illuminance meters, and luminance meters. The large dynamic range of the standard photometers eliminates the need for maintaining many working standard lamps of various wattages. Uncertainty is reduced due to a short calibration chain for working standards and simplified procedures.

The absolute integrating sphere method allows for the realization of the luminous flux unit using an integrating sphere and the calibration of standard lamps much more quickly than with a goniophotometer. This allows for the calibration of working standard lamps as well as primary standard lamps at the same time with the same uncertainty. The correction technique for the spatial nonuniformity errors in an integrating sphere is useful in reducing the calibration uncertainty of substitution measurements in an integrating sphere.

The improved photometric standards have made it possible for NIST to provide a wider variety of photometric calibration services with a wider range of calibration artifacts.

## Footnotes

1 As an aid to the reader, the appropriate coherent SI unit in which a quantity should be expressed is indicated in brackets when the quantity is first introduced.
